# Spontaneous Pneumomediastinum: A Rare Cause of Chest Pain

**DOI:** 10.7759/cureus.47015

**Published:** 2023-10-14

**Authors:** Daniela Barroso, Diana Rocha, Filipa Abelha Pereira, Rui Ribeiro, João Pimenta Fernandes, Ana Pais Monteiro, Isabel Fonseca Silva, Fabienne Gonçalves

**Affiliations:** 1 Internal Medicine, Centro Hospitalar Universitário de Santo António, Porto, PRT; 2 Nephrology, Centro Hospitalar Universitário de Santo António, Porto, PRT; 3 Medical Education, Instituto de Ciências Biomédicas Abel Salazar, Porto, PRT

**Keywords:** emphysema, chest pain, mediastinal emphysema, spontaneous pneumomediastinum, pneumomediastinum

## Abstract

Spontaneous pneumomediastinum is a rare medical condition characterized by the presence of free air in the mediastinum, not preceded by trauma, surgery, or another medical procedure. It predominantly affects young adult males and usually has a benign course, and in most cases, it is not possible to identify the precipitating factor. There are some conditions that predispose to its occurrence, namely those that lead to an increase in intrapleural pressure, such as coughing, vomiting, or vigorous exercise.

We report a case of a 21-year-old male who presented with acute-onset shortness of breath after an episode of coughing and was found to have mediastinal and subcutaneous emphysema. Clinical, laboratory, and radiological studies did not demonstrate any predisposing factor, and the case was classified as spontaneous pneumomediastinum.

## Introduction

Spontaneous pneumomediastinum, also known as mediastinal emphysema, is an uncommon condition that affects young males between the ages of 17 and 25 and is observed in approximately one out of every 30,000 hospital admissions [1,2].

Air can find its way into the mediastinum through various pathways, such as perforations in the esophagus, trachea, and bronchi [3,4]. It can also enter via the retroperitoneum after an abdominal organ has been punctured [2,3]. Additionally, air can infiltrate through wounds caused by procedures like tracheostomy or cervical and thoracic injuries, and even dental operations involving high-speed air turbine drills during tooth extraction pose a risk of introducing air into the mediastinum and causing pneumomediastinum [2,4].

The most common cause of pneumomediastinum is a non-traumatic rupture of the marginal pulmonary alveoli, allowing air to travel along interstitial and vascular routes [2]. This type is referred to as mediastinal emphysema and includes a subgroup known as spontaneous pneumomediastinum where no underlying disease is evident [2,4].

## Case presentation

A previously healthy 21-year-old male patient, with no history of trauma, drug usage, asthma, or other disease, presented to the emergency department with acute retrosternal chest pain following two days of pharyngeal odynophagia and cough. The pain irradiated towards the neck and was associated with dyspnea. He had no fever or wheezing. He denied episodes of nausea or vomiting.

On clinical examination, the patient was calm, oriented, and acyanotic, with a respiratory rate of 20 breaths per minute and a saturation of 98% on room air. His heart rate was 78 beats per minute, his blood pressure was 107/78 mmHg, and his axillary temperature was 36.8°C. He had normophonetic heart sounds with no pathological jugular turgescence, and a respiratory examination revealed bilateral vesicular breath sounds. His examination was notable for the presence of subcutaneous emphysema on the superior anterior chest wall and neck. There was no pulsus paradoxus, and Hamman’s sign was absent. The remaining physical examination was unremarkable.

On investigation, hemoglobin level was 15 g/dL (normal range: 13-17 g/dL), white blood cells were 7,100/uL (normal range: 4,500-11,000), and C-reactive protein was slightly elevated at 13.61 mg/L (normal range: 0-5 mg/L). Liver function tests, blood urea, serum creatinine, blood glucose, and serum electrolytes were normal. Despite the dyspnea, his arterial blood gas was normal, and a COVID-19 infection was excluded. The EKG showed a normocardic sinus rhythm.

**Figure 1:**
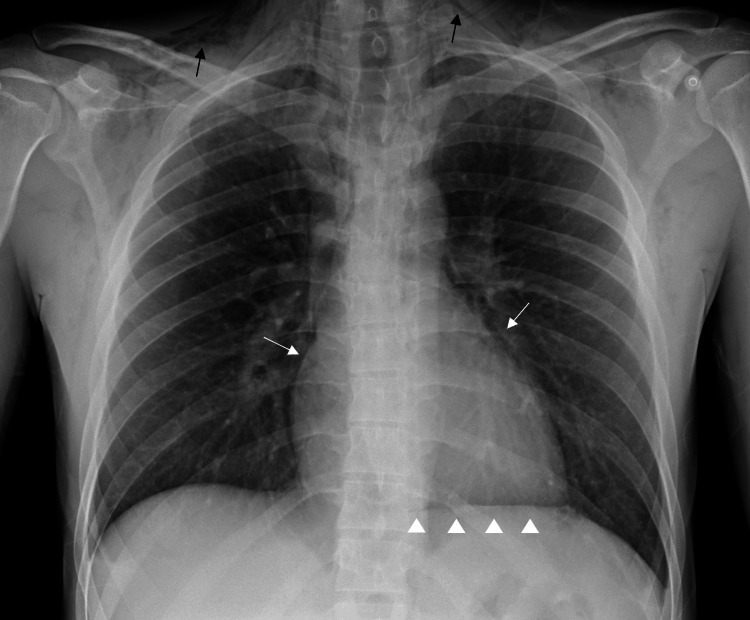
Chest radiograph An anteroposterior radiograph chest view shows a sharp line of lucency outlining the left and right heart borders (white arrows); mediastinal air between the heart and the superior surface of the diaphragm (“partial” diaphragm sign denoted by arrowheads); and subcutaneous emphysema in the supraclavicular and neck areas (black arrows).

A chest radiograph revealed a distinct line on the left edge of the heart and air following along the deep cervical planes (Figure 1).

**Figure 2:**
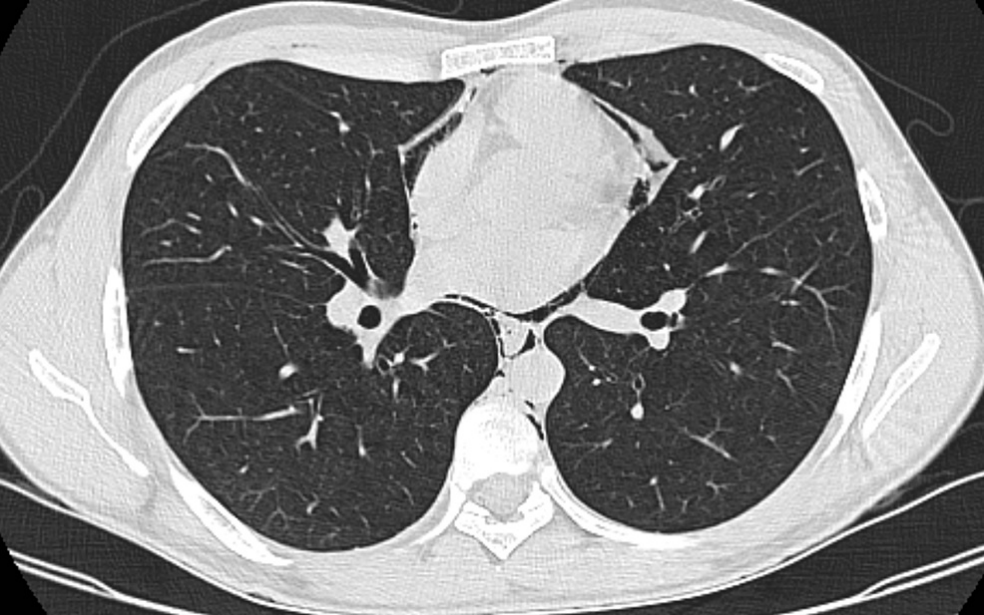
Computerized tomography (CT) scan The chest CT demonstrates the air outlining the inner surface of the mediastinal pleura and the mediastinal structures.

**Figure 3:**
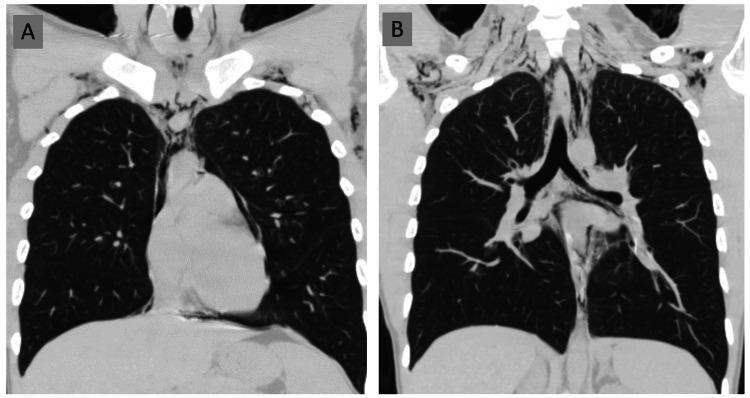
CT chest section A coronal lung window demonstrates a pneumomediastinum that extends into the superior mediastinum [A] and subcutaneous emphysema tracks along the superior aspect of the thoracic wall and neck soft tissues [B]. No pneumothorax is visible.

Further investigation with a computerized tomography (CT) scan showed pneumomediastinum with extension to the deep cervical planes and thoracic parietal emphysema without pneumothorax (Figures 2-3).

The lung parenchyma was normal, and there was no apparent discontinuity in the chest wall or airways. Infectious processes, local trauma, consumption of substances of abuse, or the presence of esophageal or tracheal foreign bodies were excluded.

The patient was admitted and treated with high oxygen therapy concentrations and analgesia while being monitored continuously. Clinical and radiological evolution were favorable, with no evidence of complications allowing discharge on the second day, with follow-up by pulmonology.

## Discussion

Spontaneous pneumomediastinum can be attributed to several factors that include rigorous physical activity, childbirth, pulmonary barotrauma resulting from deep diving, severe bouts of coughing and vomiting, asthma attacks, inhaling narcotics or those associated with bronchial asthma, and a thin biotype [1,3]. Narcotic use has been reported by some authors as the main cause of spontaneous pneumomediastinum [4,5].

The symptoms associated with this condition can vary from being completely without symptoms to severe or even life-threatening cases [1,6]. The most frequent symptom reported was chest pain, present in about 70% of the cases, and dyspnea [7,8]. Chest pain is typically located centrally and can mimic myocardial infarction or pericarditis, being exacerbated by movements, deep breathing, and relief by leaning forward while sitting [8, 9]. Other common symptoms include dysphagia and dysphonia [7-9]. On examination, it can be observed that widespread emphysema and a pulsus paradoxus, an abnormal drop in blood pressure during inhalation, or muffled cardiac sounds may also be detected [7,8].

In approximately 25% of cases, a notable, but not definitive, sign is the presence of Hamman's sign [10]. This sign is characterized by a crackling or crunching sound that aligns with the heartbeat and that can be auscultated at the sternal edge when the patient is either sitting forward or in a left lateral decubitus position [4,10].

Chest radiography continues to be the preferred method for diagnosing spontaneous pneumomediastinum [8, 11]. The accuracy of both the posteroanterior and lateral views in detecting spontaneous pneumomediastinum is nearly 100% [11].

Radiographic findings of pneumomediastinum typically involve linear gas patterns within the mediastinum, which often extend toward the cervical region [8,9]. Air bubbles or extensive air collections can be observed outlining mediastinal blood vessels, large airways, the esophagus, or even the heart, and the presence of interstitial emphysema is helpful in confirming a pneumomediastinum diagnosis. The anteroposterior chest radiograph view may show a "continuous diaphragm sign" when air is present in this space between the heart and the upper surface of the diaphragm [7,12]. Typically, the central part of the diaphragm cannot be seen in images due to its proximity to the heart, given that both structures have a similar radiological density [12]. However, when there is gas trapped between the diaphragm and the heart, it creates what is known as the continuous diaphragm sign that allows for a complete visualization of the entire diaphragm from one side to another [1,12]. Another sign to look for is known as Nacleiro's V sign, which involves gas outlining the lateral border of the descending aorta and extending laterally between the parietal pleura and medial hemidiaphragm [9,12]. Additionally, there is a distinctive finding called "the ring around the artery sign," where gas surrounds a segment of the extra pericardial region along with the right main pulmonary artery [7-9].

Pneumomediastinum can be confused with pneumopericardium, but analysis of the distribution of the gas and accompanying signs will usually distinguish these conditions [9]. The first pneumomediastinum is much more common, except in the setting of recent heart surgery, and usually manifests as a multitude of thin streaks, not confined to the region around the heart but extending to the upper mediastinum or neck [9]. Furthermore, any associated pericardial thickening or effusion (hydropneumopericardium) helps distinguish pneumopericardium from pneumomediastinum [9,11].

In cases where chest radiography is normal or inconclusive, and mostly when there is a highly suspected cause for the pneumomediastinum, performing a CT scan can be helpful [3,11,13].

When it comes to management, the focus should be on addressing the underlying cause. However, in cases where no specific cause can be identified, it is reasonable to approach treatment through observation and the use of high concentrations of oxygen with the aim of accelerating the process of natural absorption of air by the body [13,14]. To minimize the risk of deep neck space infection and mediastinitis, it is recommended to administer broad-spectrum intravenous antibiotics [13,14]. While some studies suggest the use of prophylactic antibiotics as a preventive measure against mediastinitis, more recent research has found no additional benefit [14, 15]. In fact, there have been no reported instances of patients with spontaneous pneumomediastinum developing mediastinitis [15]. In fact, Koullias et al. identified 24 patients with spontaneous pneumomediastinum, including 18 men and six women with a mean age of 17.5 years, and found that the condition responds well to conservative treatment and follows a benign natural course [11].

## Conclusions

Spontaneous pneumomediastinum is a rare and self-limiting medical condition that is more commonly seen in young adults, particularly those who engage in illegal drug use. While chest pain is the main symptom, spontaneous pneumomediastinum can present with a variety of clinical manifestations, necessitating a thorough differential diagnosis and maintaining a high level of suspicion. It is crucial to keep these factors in mind when evaluating patients with chest pain to ensure an accurate diagnosis and appropriate treatment. Chest radiography may not always provide sufficient information, and therefore, CT scans are valuable for confirming the diagnosis and evaluating any associated causes or abnormalities. Hospitalization and treatment approaches should be tailored based on individual needs, as spontaneous pneumomediastinum typically follows a benign course with a good response to conservative management.
